# The case of the mysterious pink towels

**DOI:** 10.1016/j.jdcr.2023.10.027

**Published:** 2023-12-01

**Authors:** Ross Huff, Sait Barlas, Nur Barlas, Magdy El-Din, James Toomey

**Affiliations:** a Chicago College of Osteopathic Medicine, Downers Grove, Illinois; b Florida State University Internal Medicine Residency, Lee Health, Cape Coral, Florida; c Koç University, Istanbul, Türkiye; d Al Kasr Al Ainy School of Medicine, Cairo, Egypt; e Northwestern University Medical School, Chicago, Illinois; f Department of Internal Medicine, University of Florida Health Science Center, Jacksonville, Florida; g Northwestern University, Evanston, Illinois; h Adult Infectious Diseases Division, Lee Health, Fort Myers, Florida; i Division of Infectious Diseases, Florida State University College of Medicine Internal Medicine Residency Program, Cape Coral Hospital, Lee Health, Cape Coral, Florida; j American Academy of HIV Medicine, Washington, District of Columbia; k Infectious Diseases Society of America, Arlington, Virginia

**Keywords:** apocrine, chromhidrosis, chromogenic bacteria, dermatologic infection, hygiene, mysterious, pink towels, pseudochromhidrosis, *Serratia marcescens*, towel discoloration

## Introduction

Chromhidrosis is a rare condition in which an individual’s sweat changes from colorless to having a visible color. In true chromhidrosis, this phenomenon occurs via oxidation of lipofuscin molecules excreted through apocrine and eccrine glands,^1^^,^^2^ or by the secretion of substances such as heavy metals, dyes, or systemic medications exclusively in eccrine glands.^1^^,^^3^ Pseudochromhidrosis is a more common condition through a different mechanism. In pseudochromhidrosis, the discoloration occurs as a result of the interaction between secreted sweat and an exogenous factor present on the skin, such as dyes, metals, chemicals, or chromogenic bacteria.^3^^,^^4^ A common example of this is the yellow discoloration that may occur after the use of deodorant sticks. A less commonly reported occurrence is infectious pseudochromhidrosis, which is caused by the interaction of sweat and chromogenic bacteria. This case report focuses on one such rare occurrence.

## Case report

**Fig 1:**
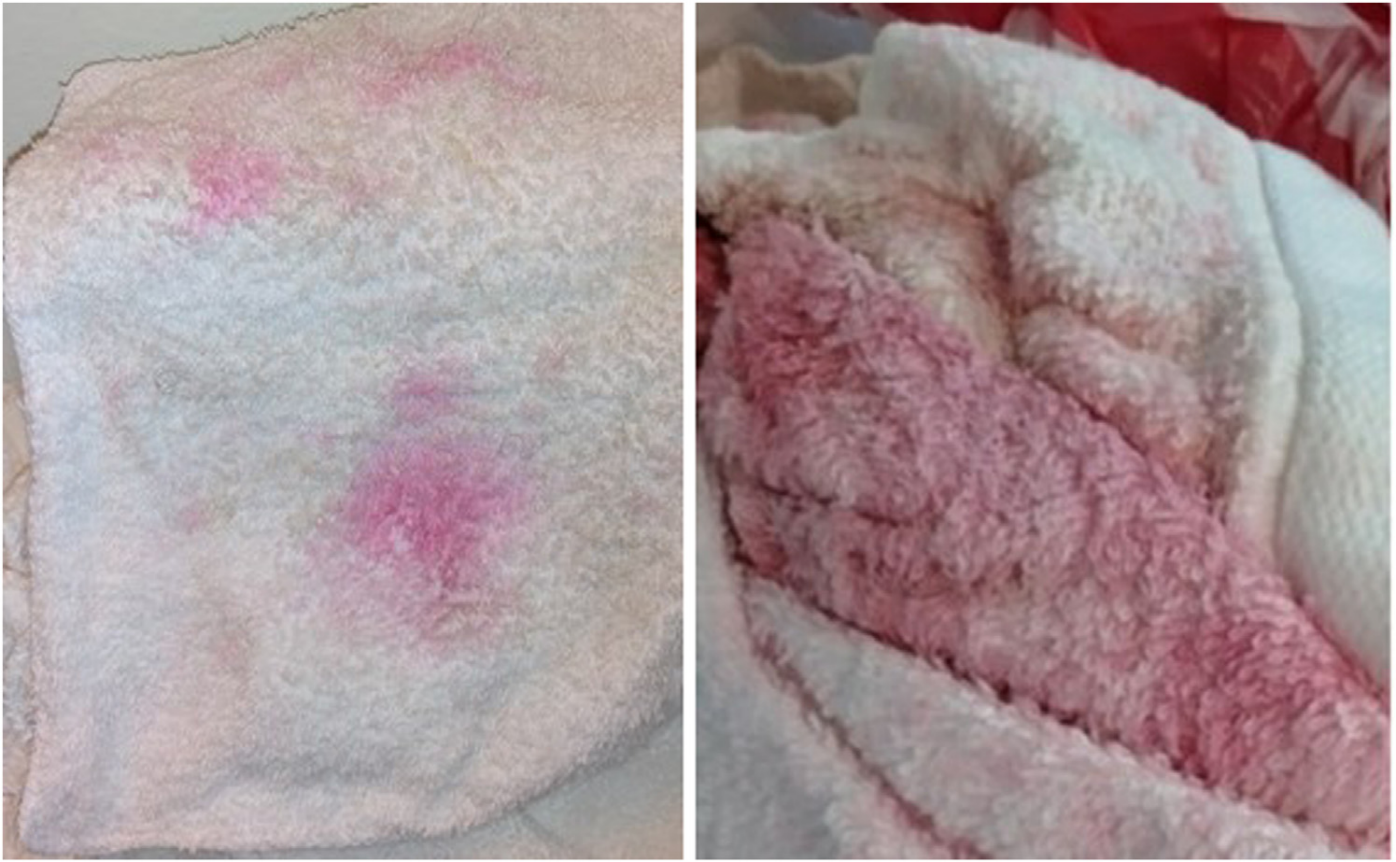
Pinkish-red discoloration present on patient’s towels. Photos taken 2 days after use.

A 50-year-old man presented to the outpatient infectious disease clinic with a 1-year history of pink discoloration of his towels 1 to 2 days after use (Fig 1). His past medical history was significant for morbid obesity, hypertension, and acid reflux. Apart from the discoloration of his towels and occasionally clothing, he had no other symptoms. Suspecting this might be stemming from environmental triggers, he had changed his personal hygiene and cleaning products over the course of the year, and replaced his shower head without resolution of symptoms. The only improvement he noted was after taking Bactrim for a prior urinary tract infection. His main concern was the strain this issue was causing on his personal relationship, as his wife no longer shared the same bathroom, would not allow him entry into her bathroom, and declined intimacy. He had been to multiple family practice physicians and emergency departments who were unable to provide answers. His visit to our clinic was the first time he was evaluated by an infectious disease physician. A thorough skin evaluation including testing patches of the skin with alcohol-based wipes showed no evidence of rashes, lesions, or discoloration. Wood lamp evaluation failed to demonstrate the presence of fungal infection or apocrine chromhidrosis. Bilateral skin cultures were obtained from the inguinal region, and the patient was provided referral to dermatology for skin biopsy. Skin culture results returned positive for heavy colonization of *Serratia marcescens*, and a diagnosis of pseudochromhidrosis was made. The patient was prescribed trimethoprim/sulfamethoxazole (Bactrim DS) twice daily for a 4 to 6 week course and instructed to wash with chlorhexidine liquid soap 3 times weekly. Although his symptoms improved with therapy, he continued to notice intermittent mild pink discoloration of his towel. Unfortunately, the patient discontinued cleansing with the chlorhexidine soap and was later lost to follow-up before further therapeutic treatments could be pursued.

## Discussion

Pseudochromhidrosis, as a result of chromogenic bacteria, is known to be caused by *Corynebacterium* spp (brown/black), *Bacillus* spp (blue), *Pseudomonas aeruginosa* (blue/green), and *S marcescens* (pink/red).^3^^,^^4^ *Serratia* causes pinkish-red discoloration through release of a red pigment called prodigiosin produced when specific conditions, including temperature and pH, are met.^5^ This pigment, among other molecules produced by the bacteria, is thought to have antibacterial, fungal, protozoal, and malarial activity, which is likely what, under the right conditions, allows the bacteria to outcompete and colonize the skin in patients afflicted. Although infectious pseudochromhidrosis does not appear to be physically dangerous, it can have a significant social and psychological impact on the patient as can be observed from our patient’s marital and mental strain.

Although literature review does not describe any occurrences of infectious pseudochromhidrosis causing frank infection, one could assume that heavy colonization with one specific bacterium may predispose the individual to future infections. There are few reported cases of infectious pseudochromhidrosis in literature and even fewer are known to be caused by *S marcescens*, which further supports that this disease is both rare and largely undetected.^6^^,^^7^ Due to limited research, there is no established treatment in literature. Combined oral and topical erythromycin has shown efficacy in treating infectious pseudochromhidrosis from *Serratia* and *Corynebacterium*. However, systemic erythromycin is a strong antibiotic regimen to start a patient on for long-term with the potential for serious side effects and antibiotic resistance.^8^, ^9^, ^10^

Our report provides an alternative therapeutic regimen using oral trimethoprim/sulfamethoxazole and topical chlorhexidine which resulted in improvement of the patient’s symptoms during the period of good adherence with the regimen. The lack of complete resolution, with persistence of intermittent mild discoloration, may have been due to nonadherence with therapy, as the patient had discontinued using chlorhexidine in the shower as previously instructed. However, it is likely not that simple. Length of treatment does appear to play a role, as skin decolonization is required to prevent recurrence of symptoms. Other factors may also have influenced the outcome, such as increased risk of bacterial skin colonization due to obesity (the patient’s body mass index was 44), or a continued (unrecognized) exposure to *Serratia* in the patient’s environment. Successful treatment of pseudochromhidrosis may depend upon a combination of therapies including topical and/or systemic antibiotics, skin decolonization, weight loss, and interruption of environmental exposure.

## Conflicts of interest

None disclosed.
